# Transtumoral approach and piecemeal resection (Steiner principle) for the treatment of tongue cancer at stage T1-T2: A pilot study

**DOI:** 10.4317/medoral.26696

**Published:** 2024-08-01

**Authors:** Kuauhyama Luna-Ortiz, Gabriela A Guillén-Hernández, Claudia Haydee Sarai Caro-Sanchez, Zelik Luna-Peteuil, Ana María Cano-Valdez, Dorian Y Garcia-Ortega

**Affiliations:** 1 partment of Head and Neck Surgery at Instituto Nacional de Cancerología, México; 2 Department of Surgery (Head and Neck) at the Hospital Manuel Gea Gonzalez, México; 3 Departmet of Pathology at Instituto Nacional de Cancerología, México; 4 Medical School at Universitatea de Medicină și Farmacie "Grigore T. Popa", Romania; 5 Department of Surgical Oncology at Instituto Nacional de Cancerología, México

## Abstract

**Background:**

To assess the feasibility of maximizing negative margins with minimal resection of healthy tissue, as confirmed by intraoperative assessment. This approach aims to be safe, effective, and to be considered a standard procedure.

**Material and Methods:**

A prospective pilot study. Peritumoral ink marking aided in identifying margins. Transtumoral incisions were made along the central line until healthy tissue was visible. If positive or close margins were identified, an extension was performed only in the involved area. The tumor bed and outer part of the tumor were inked to determine margins for intraoperative assessment of the specimen.

**Results:**

Twelve patients with oral squamous cell carcinoma participated in the study, comprising 3 men and 9 women, with a mean age of 58 years. Four patients were diagnosed with clinical stage I (T1N0), while eight were classified as stage II (T2 N0). All patients underwent ipsilateral neck dissection (levels I-III).

Intraoperative outcomes included negative, positive, or close margins. The number of tissue blocks varied based on the size of the tongue tumor and the segments that required expansion to ensure a tumor-free margin (>1 mm), which was necessary in 8 patients. All final pathological reports indicated negative margins of >1 mm.

**Conclusions:**

Piecemeal resection emerges as a feasible and oncologically sound procedure for achieving margins >1mm, which are deemed safe. Precisely identifying positive areas within the tumor proves significantly safer than *en bloc* resections. The prognoses observed in this series depended more on regional disease factors than on specific characteristics of the primary tumor.

** Key words:**Tongue, oral cavity, cancer, early stage, resection.

## Introduction

Tongue cancer accounted for 17,860 new cases and 2,790 deaths in 2022, ranking 17th in incidence and 15th in mortality worldwide, according to GLOBOCAN 2020 (included in oral cavity cancer) (1-3). Surgery remains the gold standard for early-stage tongue cancer (T1/2N0). Therefore, performing a thorough initial surgery in these patients is essential (4).

Despite advances in treatment methods and surgical techniques, patients with tongue cancer have a poor prognosis, marked by a high local recurrence rate of up to 80% within the first two years and 20% thereafter. The 5-year rates for local recurrence, regional recurrence, and distant recurrence have been reported as 11.7%, 7.8%, and 4.7%, respectively. Meanwhile, the 5-year survival rate for early stages stands at 77% (5,6).

The current surgical approach involves *en bloc* resection with macroscopic negative margins of up to 1 cm. However, there are alternative methods used in other upper aerodigestive tract sites in which transtumoral resections ensure negative surgical margins. It was once believed that transtumoral incisions could lead to local or distant spread of tumor cells. However, Steiner W. challenged this notion (6,7). The objective of our study is to assess the feasibility of maximizing negative margins with minimal resection of healthy tissue, as confirmed by intraoperative assessment. By means of extending the excision margins only at the site where positive or close margins are identified, thus avoiding the removal of large portions of the tongue. This approach aims to be safe, efficient, and to be considered a standard procedure.

## Material and Methods

- Study design

A prospective, analytical, intervention, pilot study spanning from May 2022 was approved by the Research and Ethics Committee of Instituto Nacional de Cancerología, Mexico (reference number 023/031/CCI, CEI/036/22). The study included patients over 18 years old with a histopathological diagnosis of squamous cell carcinoma in the mobile portion of the tongue, and tumor size T1, T2, N0 based on the AJCC Cancer Staging Manual, 8th Edition. Patients who had not been treated previously, underwent preoperative clinical examination, tumor biopsy, neck ultrasound, and, in case of uncertainty regarding the tumor extension, MRI or CT scans.

The study is divided into two parts. This first report focuses on patients and the surgical technique. The second report will assess long-term follow-up for potential recurrences and survival rates. Clinical follow-up will be conducted every 2 months in the first year, every 4 months in the second year, every 6 months in the subsequent 3-5 years, and then every year. If there is suspicion of local recurrence, an incisional biopsy will be performed. In cases of suspected locoregional recurrence (involving the tongue and neck lymph nodes), both an incisional biopsy and ultrasound will be performed, along with a fine-needle aspiration biopsy of the lymph nodes if malignancy is suspected.

- Description of the surgical technique

**Figure 1:**
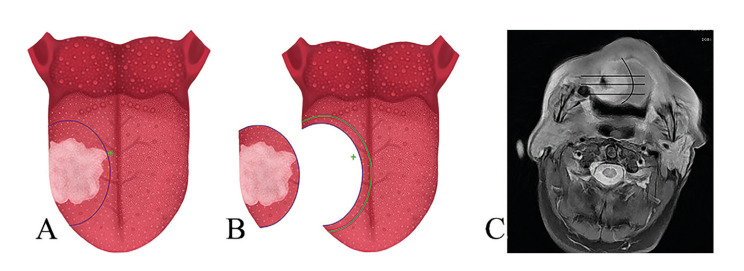
A) Resection of a tongue tumor, with the asterisk (*) indicating the area at risk of proximity to tumor margins. B) Re-resection of tumor bed with extension into the positive area, including extensive healthy tissue. C) MRI and CT scan revealing irregular tumors.

Current practice involves obtaining tissue by marking clinical margins of 1 cm and sending the specimen for final pathological examination. Margins in the posterior area carry an increased risk of being positive and often require the removal of larger portions of healthy tissue (Fig. 1). This can be confirmed using MRI and/or CT scans revealing irregular tumors (Fig. 1).

**Figure 2:**
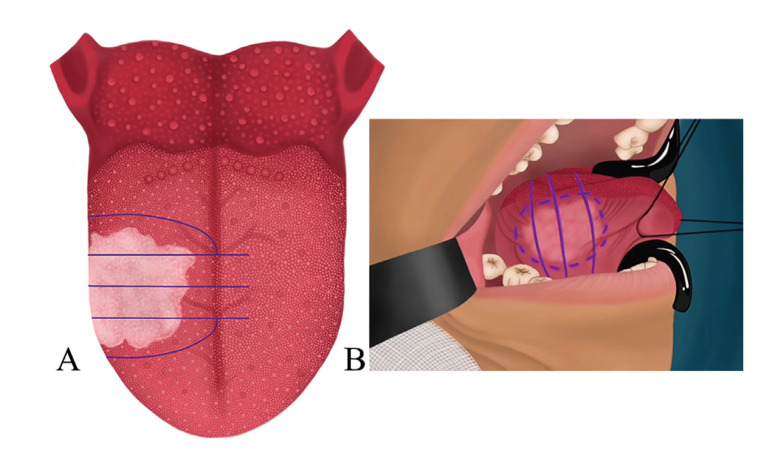
A) Planning of transtumoral piecemeal resection. B) View of the inked lateral edge, where the floor of the mouth is included as a surgical margin.

**Figure 3:**
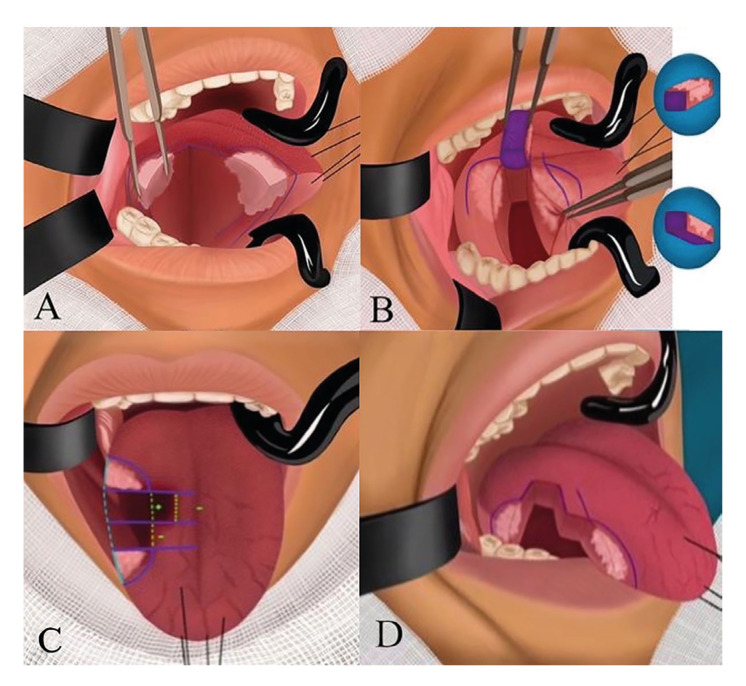
A) Transtumoral incision until healthy tissue is visible. B) Piecemeal resection, tumor bed is inked for intraoperative assessment. C) Positive tumor bed, resection is performed only on the segment closest to the positive margin. D) Lateral view of the edge of the tongue.

We carried out the procedure according to the following steps:

1. Peritumoral ink marking: The tumor is marked by palpation, simultaneously marking the margins as if conducting an *en bloc* resection (Fig. 2). However, a line is drawn extending beyond the tumor, as shown in Fig. 2, and may even encompass part of the mucosa of the floor of the mouth (Fig. 2).

2. A transtumoral incision is made through the central line until healthy tissue is visible (Fig. 3). After obtaining the central specimens, each is labeled as either anterior or posterior, and the tumor bed is inked. (Fig. 3). The lines next to the midline allow marking the resection sites. If the tumor bed is identified to be near one of them, the precise re-resection site can be located to provide an adequate margin at the site where such margin needs to be expanded (Fig. 3).

3. If there is any positive or close margin, only an extension of the margin in the

 involved tumor bed is performed (Fig. 3).

4. The edges of the tumor, along with the tumor bed and the outer part of the tumor, should be inked to provide their margins (Fig. 3).

- Intraoperative assessment of the specimen

1. Laterality is confirmed.

2. The specimen is oriented according to laterality, specifying the number of samples taken, with 1 to 3 designated, or 4 if necessary, starting from the anterior area and moving towards the posterior.

3. The fresh samples (segments) are referred to by the surgeon as superior, inferior, and the inked tumor bed, both anterior and posterior in each case.

4. Each side of the samples is photographed and measured.

5. The margins of all samples are inked as follows: tumor bed (black), superior (blue), inferior (green); in addition to the anterior edge in orange and the posterior edge in brown.

6. The presence of a visible tumor, ulcer, retraction, or induration is determined.

7. The area is measured and documented.

8. In each segment of the specimen, one or two coronal or axial cuts are made relative to each edge.

9. Each positive margin or suspicious area is measured macroscopically.

10. If any margin is less than 5 mm, a thin frozen section of the tumor and the nearest margin is prepared.

a. The tissue is previously impregnated in Tissue-Tek medium and frozen at -25 °C (Leica CM1860/CM1860UV Cryostat).

b. Sections of 4-µm are cut and stained with the usual hematoxylin and eosin method.

c. The slides are examined under the microscope to determine the distance in mm between the tumor and the inked margin.

11. The diagnosis is issued in writing in the intraoperative assessment format.

12. If necessary (margin < 2 mm), the surgeon will decide whether to forward the sample for a final report or perform an additional resection, marking the new tumor bed with ink.

13. Each segment is individually placed in formalin with its corresponding label and then undergoes the standard paraffin embedding procedure.

## Results

Over an 18-month period, we studied 12 patients with oral squamous cell carcinoma who underwent resection, including 3 men and 9 women with a mean age of 58 years (range 44-72 years). Four patients were diagnosed with clinical stage I (T1N0), while 8 were classified as stage II (T2 N0). All patients underwent ipsilateral neck dissection (levels I-III) Additionally, one patient with a central tumor underwent contralateral dissection (levels I-III). The mean operative time was 95 min (range 80-110 minutes). Patients typically stayed in hospital for 2 days (range 1-3 days). No perioperative complications were observed. Our hospital protocol does not include reconstruction when the resection involves the midline or the floor of the mouth.

The resections were performed as per the technique description. Eight patients were upstaged, while one patient was downstaged. Histological grading revealed Grade 2 (G2) in 10 cases, Grade 3 (G3) in one case, and one case of carcinoma in situ. The mean depth of invasion was 5.5 mm (range 2-16 mm). Four patients had a worst pattern of invasion score of 5. Perineural invasion was observed in six patients, whereas lymphovascular invasion was identified in three patients. Four patients received combined adjuvant therapy (chemotherapy/radiotherapy), and three patients underwent radiotherapy (Table 1).

Table 2 shows the initial status of the intraoperative assessment, indicating whether margins were negative, positive, or close. The number of blocks used depended on the extent of the tumor in the tongue and the segments that were expanded to achieve an intraoperative tumor-free margin of >1 mm. Segments were expanded in 8 patients. All final pathological reports confirmed negative margins at >1 mm. No patient has experienced recurrence thus far.

## Discussion

At the 2022 Combined Otolaryngology Spring Meetings in Dallas, Texas, topics related to tongue cancer were discussed in a plenary session. Issues such as the challenges of obtaining tumor-free margins through *en bloc* resection and concerns regarding the risks associated with extensive resections that could compromise tongue function or viability were highlighted. The tumor bed and central portion of the tumor were identified as particularly risky locations. An analysis of 20,602 cases of early-stage oral cavity cancer in the United States describes that positive margins can occur in up to 43% of cases. Tumor factors, including stage, grade, and location are indicative of the disease aggressiveness and the difficulty of resection. Additionally, factors such as surgeon’s skills, type of treatment center, hospital case volume, and geographical region contribute to the complexity of treatment (8).

In Fig. 1 we illustrate the resection of a tongue tumor, with the asterisk (*) indicating the area at risk of proximity to tumor margins. In cases where the intraoperative assessment reveals positive or close margins, a re-resection of the tumor bed, akin to the initial procedure, must be performed. This re-resection usually involves removing the entire extent of the tumor bed, which may compromise healthy tissue, as depicted in Fig. 1. Hinni ML *et al*. (9) noted that during attempts to identify positive margins, deviations of 1 cm occur. Therefore, the piecemeal technique is used to pinpoint the area of interest and the site of resection. This technique is also more conservative, as described in Fig. 3. Maxwell JH *et al*. (10) analyzed 3 groups: 1) No sampling of specimens, 2) Examination of specimens, with additional tissue obtained if positive or suboptimal, and 3) No examination of specimens, with excision expanded like group 2. Their findings suggested that conducting intraoperative assessment and expanding margins did not yield better outcomes than in group 1, possibly due to wider resections in that group. However, their study underscores the challenge of identifying the highest-risk site after extensive resections. Consequently, some authors advocate for piecemeal resection, aligning with our current practice and the technique illustrated in our study (10,11).

Even though *en bloc* resection is generally regarded as the gold standard, alternative approaches have been explored with promising results. However, many of these approaches remain controversial, particularly in anatomically constrained structures such as the larynx or skull base where piecemeal resection is often the preferred method. We propose the use of piecemeal resection in the tongue, despite its broader anatomical structure, due to the complexity of examining such extensive tissues and the challenges faced by pathologists in making accurate diagnoses during intraoperative assessment. Moreover, wider re-resections cannot be justified on these grounds. In our study, although wide multifragmented resections were initially performed, subsequent analysis revealed that 8 out of 12 patients required re-resection in specific sites, all without compromising extensive healthy tissue (Table 2).

Ex vivo tissue shrinkage is a well-documented phenomenon, with linear shrinkage ranging from 25% to 75% depending on the tissue components (12). Immersion fixation further exacerbates this shrinkage. Consequently, microscopic measurements of tissue embedded in paraffin will be lower compared to those taken from fresh tissue during intraoperative assessment.

The results regarding the relevance of shrinkage after fixation range from 32 to 42%, often resulting in closer margins than those observed in fresh tissue. This discrepancy poses challenges for achieving the margins required for a final histopathological report, where margins of 2-5 mm are considered close, and 1 cm is a macroscopic margin. The average tongue width is 5cm, while the average length is 10cm, of which 2/3 correspond to the mobile tongue, though this varies among individuals. In cases involving a 2cm T1 tumor and a 2-4 cm T2 tumor, adhering to the guidelines proposed by AJCC and NCCN would entail a loss of 50% or more. Consequently, we concur with Fowler J *et al*. (13) in questioning the feasibility of a 5 mm margin as the sole option, proposing instead that margins exceeding 1 mm be considered negative and oncologically equivalent to 5 mm. Moreover, the previously described technique facilitates achieving close margins by enabling a thorough intraoperative assessment allowing for the safe expansion of the tumor bed at an appropriate site without significant loss of tongue tissue and with similar safety for the patient (14).

Furthermore, Tasche KK *et al*. (15) did not find that additional tissue resection from the tumor bed to obtain greater margins was associated with improved local recurrence rates or overall prognosis. Determining the closeness of a margin intraoperatively would involve examining the tumor specimen itself, as per our protocol, rather than the tumor bed.

Tasche KK *et al*. (15) also addressed the definition of close margins, which, as they have shown, do not necessarily imply a worse prognosis, as opposed to positive margins, where tumor cells are present at the resection site. A margin of less than 5 mm is considered close; however, this arbitrary definition lacks supporting evidence.

The absence of a defined cutoff value for a close margin, necessary for making clinical decisions on adjuvant treatments, renders it a weak predictor of recurrence (15). According to the MSKCC series (16), close margins are defined as falling between 2.3 and 5 mm, while positive margins range from 0.01 to 2.2 mm. Tumors within the positive margin range would likely require adjuvant treatment.

At our hospital there is uncertainty regarding which patients should receive adjuvant treatment for close margins, particularly those with margins involving less than 5 mm. Therefore, we suggest conducting piecemeal resection with minimal removal of tongue tissue to avoid extensive reconstruction, until criteria to determine the appropriate candidates for adjuvant treatment are established. This approach aims to prevent the misclassification of patients identified as high risk but who are actually low risk, which could result in unnecessary overtreatment and toxicity, as suggested by Tasche KK *et al*. (15).

The final oncological outcomes in the present study had greater significance in regional disease, which shifted from being considered an early stage to an advanced stage, but not due to the margins of the primary resection.

The limitation of the present study is that there are still no long-term oncological outcomes; therefore, we only propose a surgical treatment. A larger number of patients and more time are needed to report long-term oncological outcomes.

## Conclusions

Piecemeal resection emerges as a feasible and oncologically sound procedure for achieving margins >1mm, or 2.3 mm depending on how the current evidence is interpreted, which are deemed safe. Precisely identifying positive areas within the tumor proves significantly safer than *en bloc* resections. The prognoses observed in this series depended more on regional disease factors than on specific characteristics of the primary tumor.

## Figures and Tables

**Table 1:** Histology, Staging and Final treatment.

No.	TUMOR cm	pT	pN	PS	GRADE	DOI mm	WPOI	PNI	LVI	Adjuvant
1	1.6x1.5	pT2	pN2b	IVA	G2	6	5	SI	NO	QT/RT
2	1.4x1.1	pT2	pN1	III	G2	8	3	SI	NO	QT/RT
3	3.8x1.5	pT2	pN2b	IVA	G2	8	3	SI	SI	QT/RT
4	2.2x0.7	pT2	pN1	III	G3	5	4	NO	SI	RT
5	1x1.5	pTis	pN0	IS	IN SITU	-	-	NO	NO	NO
6	3.5x3	pT2	pN2b	IVA	G2	16	5	SI	SI	RT
7	2.7x1.7	pT2	pN2b	IVA	G2	4	3	NO	NO	RT
8	1x1.5	pT1	pN0	I	G2	3	3	NO	NO	NO
9	1.5x0.8	pT1	pN0	I	G2	2	4	NO	NO	NO
10	1x0.6	pT1	pN0	I	G2	6	5	NO	NO	NO
11	1x1.5	pT1	pN2b	IVA	G2	5	5	SI	NO	QT/RT
12	1.4X1X0.5	pT1	pN1	III	G2	4	3	SI	NO	NO

**Table 2:** Transoperative study characteristics of the resection.

No.	FIRST TS	BLOCKS #	EXTRA CUTS #	EXPANDED SEGMENT
1	Negative	4	0	-
2	Positive	4	1	2,3
3	Negative	4	0	-
4	Positive	4	1	2,3
5	Negative	2	0	-
6	Nearby	4	1	2,3
7	Nearby	4	1	1,3
8	Nearby	4	1	3
9	Positive	2	1	1,2
10	Positive	3	1	1,2,3
11	Nearby	3	1	2
12	Negative	4	0	-

TS= transoperative study.
